# Extinction during memory reconsolidation blocks recovery of fear in adolescents

**DOI:** 10.1038/srep08863

**Published:** 2015-03-09

**Authors:** D. C. Johnson, B. J. Casey

**Affiliations:** 1 Weill Medical College of Cornell University, Sackler Institute for Developmental Psychobiology, New York, NY 10065, USA

## Abstract

Adolescence is a time of intensified emotional experiences, during which anxiety and stress-related disorders peak. The most effective behavioral therapies for treating these disorders share exposure-based techniques as a core component. Exposure-based therapies build on the principles of fear extinction learning and involve desensitizing the individual to cues that trigger anxiety. Yet, recent evidence shows an adolescent-specific diminished capacity to extinguish fear responses, suggesting that adolescents may respond less well to exposure-based therapies than other age groups. Here we demonstrate an alternative method for blocking the recall of fear memories in adolescents, building on principles of memory reconsolidation in adults. During memory reconsolidation, a memory that is recalled becomes labile during which time it can be updated. Prior research has shown that extinction training during memory reconsolidation attenuates the recovery of fear memory in human adults and in rodents. Using this method, we show attenuation of fear memory in adolescent humans. These findings have significant implications for treating one of the most vulnerable populations to anxiety and stress related disorders - adolescents - by optimizing exposure therapy based on principles of memory reconsolidation.

Fear is an adaptive function that allows an individual to respond appropriately to the imminent arrival of danger. For most individuals who experience such events, fear responses naturally extinguish across time as the danger diminishes. However, when the fear persists long after the danger has passed, this can lead to the development of stress and anxiety-related disorders. These disorders are the most common of all the psychiatric illnesses and frequently emerge during adolescence, often persisting into adulthood^1^, with a majority of all adults first meeting diagnostic criteria during childhood or adolescence^2^.

The most effective evidence-based behavioral treatment for anxiety and stress-related disorders is exposure-based cognitive behavioral therapy (CBT)^3^,^4^. Exposure-based CBT is based on the principles of fear extinction and involves identifying the triggers for the underlying anxiety and desensitizing the patient to these triggers with repeated exposures. Emerging evidence from both rodents and humans suggests that adolescents are less capable of extinguishing fear memories relative to younger or older animals^5^,^6^ but see Ref. 7. While the amygdala, medial prefrontal cortex and hippocampus are known to constitute a core neural circuit in fear extinction learning^8^, this circuit is functionally immature during adolescence^5^,^9^,^10^,^11^,^12^. Diminished fear extinction is thought to be a risk factor for anxiety and stress related disorders. Thus emerges the paradoxical situation in which the most common behavioral treatment approach for anxiety and stress-related disorders in adolescents is built on the same learning process that may mediate these individual's vulnerability to clinical anxiety in the first place. Together, these findings suggest that extinction-based therapies may be less effective for adolescents^13^ and that alternative or optimized behavioral treatments that bypass the need for fear regulation circuitry may be more effective.

Recent studies have shown that an alternative method for attenuation of fear memories is that of memory reconsolidation^14^,^15^,^16^,^17^. Memory reconsolidation is based on the notion that memories are dynamic rather than stable^18^,^19^. Every time a consolidated memory is retrieved, it returns to a fragile state and must restabilize again before becoming a stable memory^20^,^21^. The temporal window of increased plasticity during which a memory undergoes reconsolidation begins approximately 10 minutes after memory retrieval and continues for at least one hour^17^, presenting an opportunity during which the memory can be updated and altered. Rodent^17^,^20^ and human imaging studies^15^,^16^ suggest that reconsolidation of fear memory is primarily mediated by the amygdala, rather than prefrontal circuitry. These findings suggest a plausible way in which adolescents may be able to overcome fear memories via interventions that alter memories within the amygdala.

In the current study we used a behavioral method employed in human adults^14^,^15^,^16^ and rodents^17^,^22^ to test whether fear memories could be attenuated in human adolescents. We hypothesized that adolescents who received a reminder cue 10 minutes prior to extinction training would (1) show less fear recovery relative to adolescents who only received extinction training (no reminder cue); and (2) look similar to adults who received the reminder cue. This hypothesis was based on evidence of adolescents having diminished prefrontally-mediated extinction learning^5^,^6^ and of reconsolidation update being primarily mediated by the amygdala, rather than the prefrontal cortex^15^,^16^,^17^,^20^. 

A total of 74 subjects (36 adults, 38 adolescents; see Supplementary for subject demographics) were randomly assigned to either extinction or reconsolidation update conditions to test whether reminder of a conditioned stimulus (cue) 10 minutes prior to extinction would block the recovery of fear memory. We used a modified discrimination paradigm with partial reinforcement that took place over 3 consecutive days (Figure 1). Two distinct cues were presented within two distinct visual contexts. The visual contexts (Contexts A and B) were pictures of rooms (bedroom, kitchen) and the cues were windows within those rooms that changed in color (yellow, blue). Acquisition (experimental day 1) occurred in Context A, and extinction and re-extinction (experimental days 2 and 3, respectively) took place in Context B. This manipulation was employed in an effort to isolate responses to the cue and partially control for the effect of the acquisition context to mediate fear responses during extinction and re-extinction^23^.

During acquisition, the window would change from black to either yellow or blue. One colored window (CS+) was paired 50% of the time with a compound aversive stimulus (US) consisting of validated, negatively valenced animal pictures (IAPS)^24^ and a validated aversive sound^25^. The other colored window (CS−) was never paired with the US. Twenty-four hours later, participants underwent extinction training in which the CS+ and CS− were presented repeatedly unpaired with the US. In the reconsolidation update condition, participants were reminded of the fear memory by presentation of a single trial of the CS+ 10 minutes prior to extinction. Participants in the extinction-only condition did not receive a pre-extinction CS+ reminder.

Another 24 hours later, all participants underwent reinstatement^26^ to elicit the return of extinguished fear. One presentation of the US alone was followed by re-extinction, in which participants were presented with repeated trials of the CS+ (unpaired with the aversive stimulus) and CS−. The fear response was indexed by skin conductance response (SCR), an index of autonomic nervous system arousal^27^. At each phase of the experiment, fear responses were calculated by subtracting SCR responses to the CS− from responses to the CS+.

The results from the acquisition phase are shown in Fig. 2A. There was a main effect of stimulus type [F(1,70) = 54.78, p < .0001] indicating greater SCR to the CS+ than the CS− (t(73) = 7.52, p < .0001) across all participants and a main effect of time [F(1, 70) = 4.564, p = .036] in the mean SCR difference (CS+–CS−) score from early to late trials (Figure 2A). These results confirm that participants learned to distinguish between the CS+ (threat cue) and the CS− (safety cue). Adolescents and adults showed equivalent fear acquisition across age groups [F(1, 70) = .956, p = .33] and across experimental conditions [F(1, 70) = .23, p = .64]. Thus subsequent group effects are unlikely due to group differences in reactivity to specific stimulus categories or strength of conditioning.

Similar to our previous findings, adolescents showed diminished extinction learning over time relative to adults as indicated by an age x time interaction [F(1, 70) = 3.91, p = .05]. Post hoc t-tests revealed a significant decrease in the mean SCR difference (CS+–CS−) score from early to late trials during extinction learning for the adults [t(35) = 4.34, p < .0002] but not for the adolescents [t(37) = 1.78, p = .08] (Fig. 2B). This pattern of attenuated fear extinction learning in adolescents compared to adults is in concordance with our previous findings in humans and rodents^5^.

Following reinstatement (isolated presentation of the US), adolescents and adults who received the reminder cue 10 minutes prior to extinction learning on the previous day, showed a diminished fear response. Conversely, adolescents and adults who did not receive a reminder cue showed robust recovery of the fear memory as indicated by the main effect of experimental condition (Figure 2C) [F(1, 70) = 6.263, p < .015]. These data suggest that reinstatement of fear after extinction training can be attenuated if extinction occurs within the temporal window of reconsolidation.

We reanalyzed our data across all phases of the study by Tanner stage rather than age group and found similar results (see supplemental text). We found no significant effects of sex on any phase of the experiment (ps > .28, see supplemental text).

## Discussion

The results suggest that reconsolidation update attenuates fear recovery in adolescents similar to adult humans^14^,^15^,^16^. While extinction learning involves the encoding of a new competing memory that leaves the original fear memory intact^11^, the current results suggest that the safety information provided during post-retrieval extinction is integrated into the original fear memory altering its affective value even in adolescents who show diminished extinction learning.

These findings are promising in that they suggest fear extinction learning, a form of fear regulation dependent on strong functional connectivity between the vmPFC and the amygdala^12^, is not the only means by which adolescent humans can regulate fear. Unlike other forms of memory with more diffuse neural representations, cued fear memories are thought to be stored in the amygdala^28^. In concordance with these data, recent human fMRI studies have shown little if any involvement of the vmPFC in extinction following reconsolidation update^15^,^16^, suggesting that the vmPFC plays a diminished role in reconsolidation of fear memories in humans. These findings could explain why reconsolidation successfully blocked fear recovery in the adolescents in the present study. While this hypothesis is consistent with the human adult imaging findings^15^,^16^, further studies would be required to test the neural correlates of the behavioral effects we report here for adolescents.

**Figure 2:**
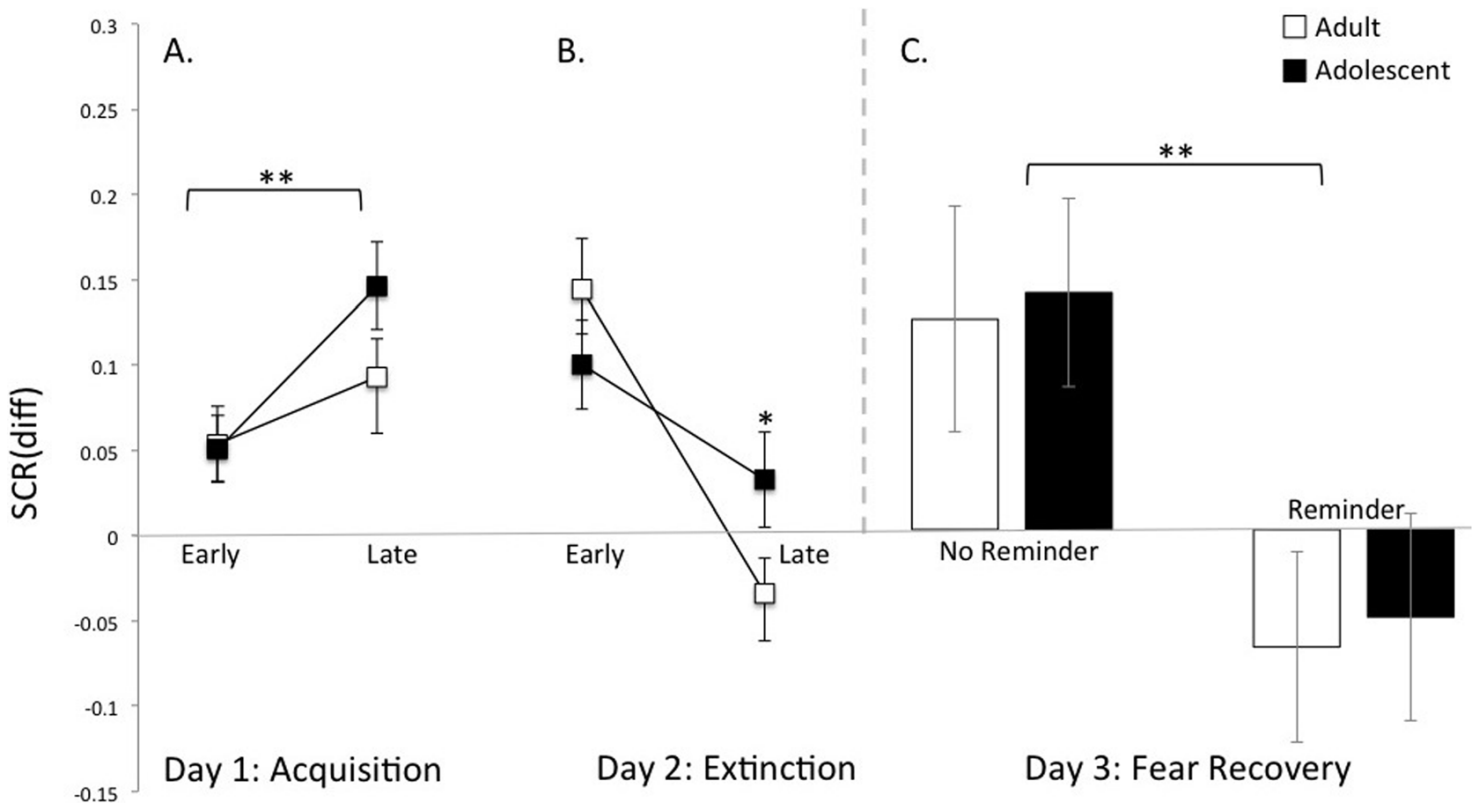
Acquisition, extinction and recovery of fear memory by age group. (a) There were no differences in differential skin conductance response (SCR) of the CS+ and CS− by age group during acquisition [F(1, 70) = .979, p = .33] and a main effect of time [F(1, 70) = 4.564, p = .036] in the mean SCR difference (CS+–CS−) score from early to late trials. (b) There was an interaction of age group x time in extinction learning as indexed by differential SCR of the CS+ and CS− [F(1, 70) = 3.913, p = .05]. Post hoc t-tests showed significant within-session extinction learning for adults (t = 4.34, p < .0002) but not for adolescents (t = 1.78, p = .08). All results are presented as a mean ± SEM. two-tailed t-test. (c) Diminished fear memory with reconsolidation update. Participants who were reminded of the conditioned stimulus 10 minutes prior to extinction showed no recovery of fear 24 hours later, as indexed by SCR responses to the first CS+ trial of re-extinction (experimental day 3). There was a main effect of experimental condition [F(1, 70) = 6.263, p = .015] and no age group x experimental condition interaction [F(1, 70) = .002, p = .966]. All results are presented as a mean ± SEM. *p = .05, **p < .05.

It should be noted that evidence of the persistence of reconsolidation update to attenuate fear, as indexed by the long-term fear recovery test (e.g. one year later^14^) is not available for the current study. Instead, we utilized reinstatement in this study, thought to be a potent assay by which to evoke the return of conditioned fear^14^. Conditioned fear did not return in the reconsolidation update group after reinstatement, even in our adolescents who showed diminished within-session extinction, highlighting the robustness of the effect. However, adolescents who showed diminished within-session fear extinction learning compared to adults and received no reminder cue, showed similar between-session extinction (fear recovery) following reinstatement as adults (Fig. 2c). It is important to distinguish between within-session extinction, which refers to decreases in fear response during the extinction session, and between-session extinction, which refers to the retention of that extinction learning when presented with the same CS at a later occasion (usually 24 hours). Between-session extinction may map more directly onto clinical models of relapse after exposure therapy than within-session extinction^29^. Reinstatement may not be the ideal assay to test for developmental or clinical differences in between-session extinction retention. Future studies that test adolescent-specific differences in between-session extinction using spontaneous recovery (passage of time) rather than reinstatement (single or multiple presentation of the unconditioned stimulus prior to the fear recovery test) to evoke the return of fear would help to constrain our findings.

Although exposure-based therapies are effective in the treatment of anxiety and stress disorders, it has been noted that as many as 40–50% of young people fail to fully benefit from such therapies^30^. The efficacy of these treatments may be impacted by age and type of therapy employed. Our data highlight how modifying the timing of therapeutic sessions based on principles of memory reconsolidation could lead to more effective attenuation of conditioned fear in both adolescents and adults. A modified version of an exposure-based CBT protocol based on memory reconsolidation might involve reminding patients of why they are there when they first arrive at the clinician's office (i.e., reminder cue), then establishing a safe and positive rapport for approximately 10 minutes (i.e., waiting for reconsolidation window) before initiating desensitization with exposure therapy. The findings may also explain why exposure-based CBT is effective for some patients and not others, and for some clinicians more than others. It is possible that positive treatment outcomes, in some cases, have been achieved through modified exposure-based CBT protocols that inadvertently capitalized on the principles of memory reconsolidation. These data provide evidence-based support for this approach. Validating such protocols would be an important next step in establishing the utility of modified clinical therapies for adolescents and adults based on the principles of memory reconsolidation.

## Methods Summary

Participants were recruited by flyer and during public events, as well as through select websites. All recruitment outlets were approved by the Weill Cornell Medical College Institutional Review Board (WCMC-IRB). All participants signed Informed Consent documents approved by the WCMC-IRB. Participants were informed that they could terminate their participation in the study at any time, for any reason, if they wished to do so. All methods were carried out in accordance with an experimental protocol approved by the WCMC-IRB.

We utilized a differential fear-conditioning paradigm with partial reinforcement that took place over three days in two different visual contexts. Visual contexts consisted of two different scenes, a bedroom and a kitchen, and conditioned stimuli consisted of two colored windows (blue and yellow) embedded within each visual context. The unconditioned stimulus was a hybrid consisting of validated, negatively valenced animal pictures (IAPS)^24^ and a validated aversive sound^25^. The measure of fear was differential (CS+–CS−) skin conductance response. Prior to starting the experiment, participants were randomly assigned to either the reminder (reconsolidation update) or no reminder (extinction) conditions.

On experimental day 1, one colored shape (the CS+) was paired on half of the trials with a compound aversive stimulus (unconditioned stimulus, CS + US) within Context A. The US consisted of an aversive sound presented simultaneously with an aversive picture from the International Affective Picture Series (IAPS)^24^. Each presentation of the US consisted of the same sound and a different picture. The other colored shape (CS−) was never followed by the aversive stimulus. Participants were presented with 32 trials on Day 1 (8 CS + US, 8 CS+, 16 CS−). Twenty-four hours later, participants returned for experimental day 2. Participants in the extinction condition started with a 10-minute rest period, in front of the test computer. This was followed by a 32-trial extinction session (16 CS+, 16 CS−) in Context B. Participants who were assigned to the reconsolidation update condition received a single presentation of the conditioned stimulus (in context B), unpaired with the aversive stimulus, prior to the 10-minute rest period. These participants received one less CS+ trial during the extinction session (15 CS+, 16 CS−) in order to match the total number of CS+ trials across experimental conditions. All participants viewed a cartoon video of Tom and Jerry (Warner Brothers) during the 10-minute break, presented on the same computer screen on which they viewed the experiment. Twenty-four hours later, participants returned for experimental day 3. Participants were instructed similarly as they were prior to experimental day 2. Participants then received a single presentation of the US, unpaired with the conditioned stimulus (reinstatement). This was followed by a 32-trial re-extinction session (16 CS+, 16 CS−).

## Author Contributions

D.C.J. and B.J.C. designed the study, analyzed the data, discussed the results and wrote the manuscript. D.C.J. collected the data.

## Supplementary Material

Supplementary Information Supplementary Information

## Figures and Tables

**Figure 1:**
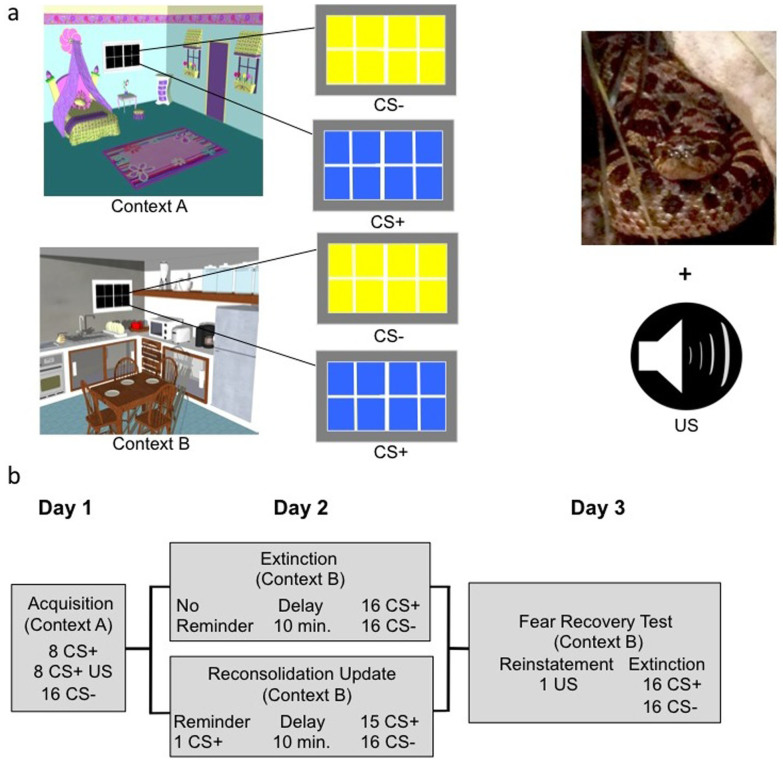
Experimental stimuli, design and timeline. (a) Contexts (A & B) were pictures of one of two rooms (kitchen, child's room) appearing on the computer screen. Conditioned stimuli (CS− & CS+) were yellow and blue windows (counterbalanced) in the rooms. The unconditioned stimulus (US) was a hybrid with visual (scary animal picture) and auditory (aversive noise) components. (b) Participants underwent acquisition on experimental day 1, extinction or reconsolidation update on experimental day 2 and fear recovery test (re-extinction) on experimental day 3 (All images by D.C.J).
