# Supplementary material for: Extinction during memory reconsolidation blocks recovery of fear in adolescents
Source: Sci Rep. 2015 Mar 9;5:8863. doi: 10.1038/srep08863 (PMC4352863; doi:10.1038/srep08863)
Supplement: Supplementary Information [file srep08863-s1.doc]

Title: Extinction during memory reconsolidation blocks recovery of fear in adolescents

Authors: D.C. Johnson, BJ Casey

Author’s affiliations: Weill Medical College of Cornell University, Sackler Institute for Developmental Psychobiology, New York, NY 10065, USA

Corresponding authors’ email: D.C. Johnson, dcj2002@med.cornell.edu; BJ Casey, bjc2002@med.cornell.edu

Supplementary Information

Participants, Full Methods and Additional Statistics

Participants.

Seventy-four of 128 participants completed the study including 38 adolescents aged 12 to 17 (extinction condition: n=19 (11f), mean age = 14.6, sd = 1.8, mean Tanner staging = 3.98, sd = .87, range 2-5; reconsolidation condition: n=19 (9f), mean age = 14.7. sd=1.6, mean Tanner staging = 3.89, sd = .56, range = 2-5) and 36 adults aged 18 to 32 (extinction condition: n = 18 (9f), mean age = 24.7, sd = 3.6; reconsolidation condition: n = 18 (9f), mean age = 24.4, sd = 4.3.) The two adolescent experimental groups did not differ in pubertal development as indexed by Tanner pubertal staging (Fisher Exact Test, p = .09). Seven of the adolescents had a Tanner stage of 5 (Extinction group: 6; reconsolidation update group: 1). The distribution of the sexes by age group and experimental condition were: Adult, extinction: n=18 (9f); Adult, reconsolidation: n = 18 (8f); Adolescent, extinction: n=19 (11f); Adolescent, reconsolidation: n = 19 (9f). Fifty-four (54) participants (33 adults, 21 adolescents) were excluded from analysis because they failed to show a reliable SCR and/or fear acquisition (magnitude of SCR to the CS+ was not greater than responses to the CS- during acquisition or during the first block of extinction (n = 47, 30 adults, 17 adolescents) or they failed to complete the 3-day study due to attrition (n = 7, 3 adults, 4 adolescents)). All participants were screened for exclusionary criteria, including hearing impairment, color blindness, diagnosed animal phobias and neurological and psychiatric disorders, as well as provided written consent prior to the experiment. Trait anxiety was measured by using the Spielberg State-Trait Anxiety Inventory Trait subscale (STAI-T)^31^ in light of evidence suggesting a negative correlation between trait anxiety and fear extinction learning^32, 33^. Trait anxiety ratings were not available for 3 adolescent participants. There was no effect of age group [F (1, 67) = .13, p =.71], experimental condition [F (1, 67) = .03, p = .86] or interaction between age group and experimental condition [F (1, 67) = 2.73, p = .10] on trait anxiety. Pubertal development for adolescent participants was measured by self-report or parental report using two standardized scales^34, 35^. Tanner staging correspondence ranged from 2 to 5 (mean = 3.9, sd = .72). The two adolescent experimental groups did not differ in pubertal development (Experimental group: mean = 3.98, sd = .87, range = 2 - 5; Reconsolidation update group: mean = 3.89, sd = .56, range = 2-5) All three sessions (days) of the experiment occurred within two hours of the original testing session for each participant. Participants were tested between 10 AM to 6 PM. There were no significant differences in time of day at which experiments were performed by experimental condition [F (1, 73) = 1.055, p=.426], age group [F (1, 73)= 1.456, p = .128], nor for age by experimental group [F (1, 70) = .307, p=.581].

Pre-study information.

Participants were not given information regarding the goals of the study or the CS-US contingencies. They were told that they might hear a loud noise and see a scary animal picture. Prior to giving consent, participants (and minor’s parents or guardians) were exposed to the pictures and sound they would see and hear during the experiment. The auditory component of the aversive stimulus was a custom-designed hybrid of white noise and a 1000-Hz tone with a duration of one second, intensity tiered for smooth onset and offset. This auditory stimulus has been previously validated as aversive^24, 36^. Sound level was individually calibrated for each participant, with participants asked to choose a volume level “loud enough that it was annoying but not so loud that it caused any pain or discomfort”. We played the sound once at a level of 95 dB and had participants rate the sound on a scale of 1 to 20 for “how annoying it was” and to ensure it was not causing discomfort. If participants rated it 15 or higher, we did not play the sound again. If they rated it less than 15, we increased the sound by at least 1 but not more than 3 dB and had them repeat their rating. We repeated this procedure one more time if they still rated it below 15, but did not repeat it after the third test trial to minimize habituation. The sound was delivered through headphones to both ears. Decibel levels were recorded for all participants. Sound intensity level ranged from 94 dB to 102 dB (mean = 98.4, sd = 2.4, n = 36) in adolescents and 87 dB to 107 dB (mean = 101.3, sd = 4.5, n = 36) in adults (t(72) = 3.42, p < .001, sound intensity levels were not available for two adolescent participants). Sound intensity level did not impact acquisition by age or experimental group, see Physiological Measurement and Analysis: Acquisition.

Experimental Design.

While several studies have demonstrated that extinction during reconsolidation attenuates conditioned fear^13, 14, 15, 37, 38^, some studies have failed to replicate these results^39, 40, 41^. One possible explanation for these discrepancies is that methodological factors play a significant role in whether reconsolidation update can be induced. As such, we implemented a design based on the studies that first reported this effect in adult humans^13^. An additional methodological consideration was that it was important to establish equivalent acquisition of conditioned fear in adolescents and adults to rule out differences in learning as a possible explanation for any observed group differences. While there is some evidence suggesting that adolescents show heightened fear conditioning compared to adults in both humans^42^ and rodents^43, 44, 45^, these findings stand in contrast to studies showing equivalent fear learning across age in humans^5^ and rodents^5, 46^. Thus components of the current design were derived from our prior work demonstrating equivalent fear learning across adolescent and adult populations^5^.

We utilized a differential fear-conditioning paradigm with partial reinforcement that took place over three days in two different visual contexts. Visual contexts consisted of two different scenes, a bedroom and a kitchen, created using 3D design software (Google Sketch-Up 2008, Mountain View, CA). Conditioned stimuli consisted of two colored windows (blue and yellow) embedded within each visual context. The unconditioned stimulus was a hybrid consisting of validated, negatively valenced animal pictures (International Affective Picture Series; IAPS)^23^ and a validated aversive sound^24^. The IAPS pictures used as UCSs were of threatening animals (IMG #s 1052 (fanged snake), 1120 (fanged snake), 1200 (spider), 1201 (spider on shoulder), 1205 (spider), 1300 (snarling dog), 1302 (snarling dog), 1932 (shark)). Visual contexts (Context A and B), stimuli and script orders were counterbalanced across subjects. Prior to starting the experiment, participants were randomly assigned to either the reminder (reconsolidation update) or no reminder (extinction) conditions and told they should “pay close attention to everything they see and hear and understand the relationship between all of these things.”

For each experimental session, stimuli were presented in a pseudo-randomized order, defined by no consecutive CS+USs during conditioning and no more than three consecutive squares of the same color in any session. Acquisition, extinction and re-extinction were each run as one continuous session, lasting 12m, 24s (conditioning, experimental day 1) and 12m, 16s (extinction and re-extinction, experimental days 2 and 3). For the acquisition phase of the experiment, the context was presented for 3s (with a black background in the window frame where the cue will be presented), followed by stimulus presentation for 7s and presentation of the US for 1s. The timing of the trials for the extinction and re-extinction phases was identical to that of the acquisition phase with the exception of no presentation of the CS+US. The intertrial interval (ITI) for all phases was 13s, providing sufficient time for SCR to return to baseline.

Acquisition (Experimental Day 1)

On experimental day 1, one colored shape (the CS+) was paired on 50% of the trials with a compound aversive stimulus (unconditioned stimulus, CS+US) within Context A. The US consisted of an aversive sound presented simultaneously with an aversive picture. Each presentation of the US consisted of the same sound and a different picture. The other colored shape (CS-) was never followed by the aversive stimulus. Partially pairing the CS+ with the tone allowed us to isolate and analyze responses to the CS+ independent of responses to the US. Participants were presented with 32 trials on Day 1 (8 CS+US, 8 CS+, 16 CS-).

Extinction (Experimental Day 2)

Twenty-four hours later, participants returned for experimental day 2. Prior to starting, participants were instructed they “may see scary pictures and/or hear annoying sounds again” and reminded to again “pay close attention to everything they see and hear.” Participants in the extinction condition started with a 10-minute rest period, in front of the test computer. This was followed by a 32-trial extinction session (16 CS+, 16 CS-) in Context B. Participants who were assigned to the reconsolidation update condition received a single presentation of the conditioned stimulus (in context B), unpaired with the aversive stimulus, prior to the 10-minute rest period. These participants received one less CS+ trial during the extinction session (15 CS+, 16 CS-) in order to match the total number of CS+ trials across experimental conditions. All participants viewed a cartoon video of Tom and Jerry (Warner Brothers) during the 10-minute break, presented on the same computer screen on which they viewed the experiment.

Re-extinction/ Fear Recovery Test (Experimental Day 3)

Twenty-four hours later, participants returned for experimental day 3. Participants were instructed similarly as they were prior to experimental day 2. Participants then received a single presentation of the US, unpaired with the conditioned stimulus (reinstatement). This was followed by a 32-trial re-extinction session (16 CS+, 16 CS-). The first 2 trials were either CS+, CS- (script order 1) or CS-, CS+ (script order 2).

Physiological Measurement and Analysis

Skin conductance response (SCR) was acquired using disposable snap electrodes pre-gelled with isotonic gel, which were attached to the distal phalanx of the second and third digits of the left hand. The signal was recorded and amplified using a skin conductance recording system (MP35; Biopac) in combination with AcqKnowledge software (Biopac). E-prime software (Psychology Software Tools) was used to control the presentation of visual and auditory stimuli and send time markers to the skin conductance recording system for each context and stimulus onset/offset. The SCR was sampled at 200 Hz with a 1-HZ filter applied. SCR was analyzed manually. For each individual subject, data were smoothed. Measurable peaks were identified as the first SCR response that occurred within .5-4.5 s following stimulus onset as defined by the difference between trough and peak being equal to or greater than .02 uS (microsiemens)^13^. A zero value was added into the analysis when no peak was detected. SCR scores were square root-transformed to normalize the distribution and were then scaled to the participant’s largest response to the CS+US during acquisition to normalize responses across participants. These SCR scores were averaged for each participant for each stimulus type separately. All SCR responses reported reflect differences between responses to the CS+ and corresponding CS-.

Acquisition. Differential fear acquisition was assessed by comparing CS+ to CS- responses across the conditioning session on experimental day 1. Only participants who showed a reliable SCR and successful acquisition of fear (i.e., SCR to CS+ greater than to CS-) were included in the analysis (n=74). We tested for age group (adolescent vs adult) and experimental group (extinction vs reconsolidation update) differences in decibel level of the auditory component of the US during acquisition with a two-way analysis of variance (ANOVA). While there was no effect of experimental group [F (1, 68) = 3.02, p = .09] and no interaction between age group and experimental group [F (1, 68) = 1.99, p = .16], there was a main effect of age [F (1, 68) = 12.18, p = .001]. Post hoc t-tests revealed the decibel level of the auditory component of the US was significantly higher for adults compared to adolescents (t(72) = 3.42, p < .001). Additional analyses were performed to determine if differences in sound intensity influenced acquisition by age or experimental groups. First, we assessed group differences in SCR to the US during acquisition by a two-way ANOVA with main effects of age group and experimental group. There was no effect of age group [F (1, 70) = .41, p = .52] or experimental group [F (1, 70) = .60, p =.44] and no age group x experimental group interaction [F (1, 70) = .36, p = .55]. Next, we examined SCR to the CS+ (unpaired with the US) during acquisition and found no significant differences in strength of acquisition by age group [F(1, 70 = .96, p=.33] or experimental group [F(1, 70 = .23, p=.64)], nor any significant age group x experimental group interaction [F(1, 70) = .08, p=.79)]. Finally, there was no correlation between decibel level and strength of acquisition (r = .07, p = .53). It is also important to note that the aversive sound constituted only one component of the compound aversive stimulus, which also included a visual component that was held constant across all subjects.

We also assessed acquisition by Tanner staging group and sex, as indexed by SCR to the CS+ (unpaired with the US) during the acquisition session. There was no effect of Tanner staging group on acquisition [F (1, 73) = .54, p = .463] and no significant effects of sex on acquisition [[F (1, 73) = .319, p = .57].

Extinction. We analyzed extinction learning by using a mixed effects three-way ANOVA with experimental condition and age group as between-subject factors and time as a within-subject factor (early and late blocks of extinction, as indexed by responses to the first five and last five trials of extinction). There was a main effect of time [F (1,70) = 19.234, p < .0001] and an interaction of age x time [F (1, 70) = 3.913, p = .05], and no interaction between time x experimental group [F (1, 70) = 1.467, p = .23] nor interaction of time x age group x experimental group [F (1, 70) = .005, p =.95]. There were no significant effects of sex on extinction learning [F (1, 73) = .0003, p = .98]. Replacing age group with Tanner pubertal staging group resulted in a significant pattern of extinction learning (F (1, 73) = 5.193, p = .03) with participants between Tanner stages 2 and 5 (n = 31) showing less extinction learning than participants at Tanner stage 5 (n = 43).

Fear Recovery. Fear recovery on experimental day 3 was calculated by subtracting SCRs from the last CS+ of extinction to the first CS+ of re-extinction. Fear recovery was assessed by a two-way ANOVA with main effects of experimental group and age group on differential SCR responses to the first CS+ presentation during re-extinction minus the last CS+ trial of extinction. There was no effect of age group [F(1, 70) = .296, p = .59] and no age x experimental group interaction [F(1, 70) = .163, p = .69], only the main effect of experimental group [F(1, 70) = 11.72, p = .001]. There were no significant effects of sex on fear recovery in either the extinction [F (1, 36) = .067, p = .79] or reconsolidation update [F(1, 36) = 1.164, p =.29] conditions. Furthermore, there was no significant effect of Tanner staging group on fear recovery in either the extinction [F (1, 36) = .574, p =.45] or reconsolidation update [F (1, 36) = 1.654, p = .21] groups. Finally, we showed no effect of stimulus order on fear recovery for either the extinction [F (1, 36) = .08, p = .778] or reconsolidation [F (1, 36) = .053, p = .82] conditions.

We additionally examined fear recovery on experimental day 3 by testing only the first CS+ - CS+ trial during re-extinction. Fear recovery was assessed with a two-way ANOVA with main effects of experimental group and age group on differential SCR responses to the first CS+ presentation during extinction. There was no effect of age group [F(1, 70) = .1 p =.75] and no age x experimental group interaction [F(1, 70) = .002, p = .97], only the main effect of experimental group [F (1, 70) = 6.263, p = .015].

Furthermore, there was no significant effect of sex on fear recovery in either the extinction [F (1, 36) = .035, p = .85] or reconsolidation update [F(1, 36) = .417, p=.52]. Furthermore, there was no effect of Tanner staging group on fear recovery in either the extinction [F(1, 36) = .243, p = .63] or reconsolidation update [F(1, 36) = .067, p = .8]. Finally, we showed no effect of stimulus order on fear recovery for either the extinction [F (1, 36) = 1.18, p = .28] or reconsolidation [F (1, 36) = .316, p = .58] conditions.

We tested the effect of age on fear recovery in the no-reminder group during re-extinction. There was no significant effect of age on SCR to the CS+ [F(1, 36) = .145, p=.706, S1A) nor to the differential CS+ (CS+ minus CS-) [F(1, 36) = .022, p = .882, S1B] during the first trial of re-extinction on day 3 or in the differential CS+ (CS+ minus CS-) during the first trial of re-extinction of day 3 relative to the last trial of extinction on day 2 [F(1, 36) = .361, p = .552] (Figure S1C).

Finally, there was no effect of stimulus order on fear recovery for either the extinction [F (1, 36) = .08, p = .78] or reconsolidation update [F (1, 36) = .053, p = .82] groups (Adult, extinction, version 1, n=10; Adult, reconsolidation update, version 1, n=10; Adolescent, extinction, version 1, n=10; Adolescent, reconsolidation update, version 1, n=9).

Post hoc t-tests were performed to explore directional effects. An alpha level of .05 was used for all statistical tests. Statistical analyses were all performed using SPSS v. 20.0 (IBM Corp., Armonk, NY, USA).

Supplementary Figures


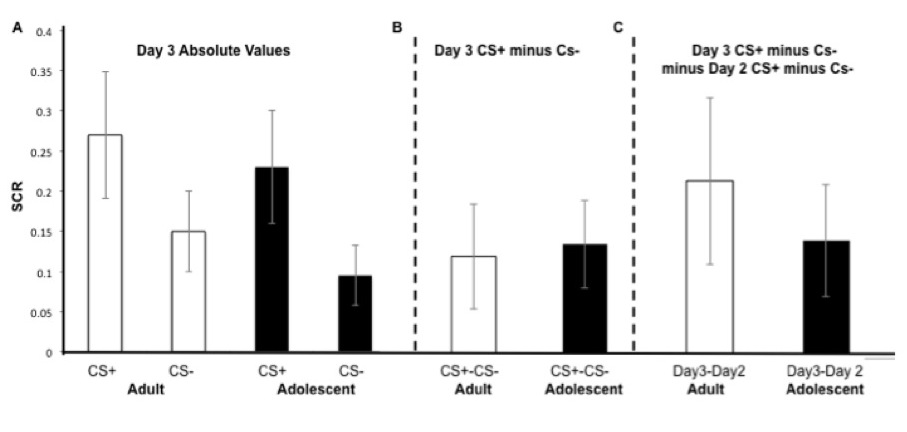


Figure S1. Fear recovery for participants in the no reminder condition by age.

Supplementary References

31. Spielberger, C.D. *et al.* *Manual for the State-Trait Anxiety Inventory* (Consulting Psychologists Press, Palo Alto, 1983).

32. Indovina, I. *et al*. Fear-conditioning mechanisms associated with trait vulnerability to anxiety in humans. *Neuron* **69**, 563-571 (2011).

33. Lissek, S. *et al*. Classical fear conditioning in the anxiety disorders: A meta-analysis. *Beh Res and Ther* **43**, 1391–1424 (2005).

34. Petersen, A.C., Crockett, L., Richards, M. & Boxer, A. A self-report measure of pubertal status: Lessons from research on anxiety. *J Youth Adol* **17**, 117–133 (1988).

35. Tanner, J.M. *Growth at Adolescence* (Blackwell Scientific Publications, Oxford, 1962).

36. Fanselow, M.S. Conditioned and unconditional components of post-shock

freezing. *Pav J Bio Sci.* **15**, 177–182 (1980).

37. Oyarzun, J.P. *et al*. Updating fearful memories with extinction training during reconsolidation: A human study using auditory aversive stimuli. *PLOS One* **7,** e38849 (2012).

38. Liu, J. *et al*. An unconditioned stimulus retrieval extinction procedure to prevent the return of fear memory. *Bio Psych.* **76**, 895-901 (2014).

39. Soeter, M., & Kindt, M. Disrupting reconsolidation: pharmacological and behavioral manipulations. *Learn Mem* **18**, 357-366 (2011).

40. Kindt, M., & Soeter, M. Reconsolidation in a human fear conditioning study: a test of extinction as updating mechanism. *Bio Psych* **92**, 43-50 (2013).

41. Golkar, A., Bellander, M., Olsson, A., & Öhman, A. Are fear memories erasable?–reconsolidation of learned fear with fear-relevant and fear-irrelevant stimuli. *Front Beh Neuro* **6** (2012).

42. Lau, J. Y. *et al*. Distinct neural signatures of threat learning in adolescents and adults. *Proc Nat Acad Sci* **108**, 4500-4505 (2011).

43. Hefner, K., & Holmes, A. Ontogeny of fear-, anxiety-and depression-related behavior across adolescence in C57BL/6J mice. *Beh Brain Res* **176**, 210-215 (2007).

44. Ito, W., Pan, B-X., Yang, C., Thakur, S., & Morozov, A. Enhanced generalization of auditory conditioned fear in juvenile mice. *Learn Mem* **16**, 187–192 (2009).

45. Den, M. L., & Richardson, R. Enhanced sensitivity to learning fearful associations during adolescence. *Neuro Learn Mem* **104**, 92-102 (2013).

46. McCallum J., Kim J.H. & Richardson, R. Impaired extinction retention in adolescent rats: effects of D-cycloserine. *Neuropsychopharmacology* **35**, 2134-2142 (2010).
